# The Philosophy of Mystery

**Published:** 1841-07-01

**Authors:** 


THE PHILOSOPHY OF MYSTERY. By *Walter Cooper Dendy*, Senior Surgeon of the Royal Infirmary for Children, &c. &c. Octavo, pp. 434. Longman & Co. April 1841.

WE will venture to aver that so curious and amusing a book has never issued from the *medical* press of this or any other country. It reminds us in every page of the erudite Burton, whose "ANATOMY OF MELANCHOLY" is so well calculated to drive away the vapours from the most confirmed hypochondriac. The research displayed in this volume is so varied and extensive, that we are absolutely astonished how a young medical man in full practice could have spared time from his professional studies and labours for the construction of a work exploring almost every labyrinth in the boundless fields of metaphysics, besides the dark paths of psychology, physiology, ideology, from the mysterious creation of man himself, down to the mummery of MESMERISM and the "OCCULT SCIENCES." We do not maintain that Mr. Dendy has unravelled all the mysteries which he has discussed; but, by throwing the dialogue into the mouths of four personages (Castally, Evelyn, Ida, and Astrophel)—two females and two males—one of them a learned student in black-letter folios, he has contrived to mingle interrogatory, opposition, scepticism, superstition, and ingenious explanation, in a spirited and amusing conversation, into which is infused a prodigious mass of the curiosities of mystery collected, one would suppose, from every work and page in the library of the British Museum! Such a work as much defies analysis as the "Anatomy of Melancholy" itself, or the large edition of Johnson's Dictionary, with its endless quotations from all the English authors that ever wielded a pen. It is pretty evident that, under the name and character of Evelyn, the author personifies himself, and enunciates his own opinions. But still it is impossible to cut out and isolate this interlocutor, without destroying the whole spirit of the dialogue. The work must be perused—and it will be extensively read both within and beyond the pale of the profession—before any conception can be formed of its nature, contents, or execution. All we can do is, to introduce a short dialogue, taken almost at random, as a mere specimen of the manner in which Mr. Dendy handles his subjects.

"ASTROPHEL. Then there is some truth in the whimsical localities in the 'Anatomy of Melancholy,' and the pictures of the tenants and apartments of the brain in the ingenious romance of the 'Purple Island' of Fletcher.

EVELYN. Although I grant that these eccentric writers evince much reading, I am not sure that their impersonations (like the 'Polyolbion' of Drayton,) do not tend to confuse, rather than elucidate, a natural subject.

Of a plurality of organs in the brain, I have been convinced, even from my own knowledge and dissections. I have seen that very considerable portions of the *cerebrum* may be removed, the individual still existing. The *vital* functions may continue, the *animal* functions are deranged or lost. The most extensive injuries of the brain, too, are often discovered, which were not even suspected; and the converse of this is often observed,—the diseases of the brain being commonly found in an *inverse ratio* to the severity of the symptoms. When *chronic* tumours and *cysts* of water are *gradually* formed, the extreme danger is averted by the balancing power of the circulation of the brain's blood; without which its incompressibility would subject it to constant injury.

In *tubercles* of the brain, it is curious that *memory* is the faculty chiefly influenced; it is sometimes rendered dull, while the fancy is vivid,—often more perfect and retentive.

Brain, however, can no more be considered as mind itself, than *retina* sight, or than the sealing-wax can be identical with the electricity residing in it. For if we look at the brain of a brute, we see how closely it resembles our own; then, if we reflect on human intellect and brute instinct, we must all believe at once that there is some diviner thing breathed into us than the *anima brutorum* of Aristotle, something more than the mere vitality,—

'Spiritus intus alit, totamque infusa per artus
Mens agitat molem.'

Brain is therefore the *habitat* of mind, the workings of which cannot be indicated without it; for, as the material world would be intact without a sense, so there can be no moral evidence of mind without a brain, which is indeed the sense of the spirit. Thus, without adopting the creed of the *Hyloist*, the *moderate* materialist,—that the mind cannot have, during the life of the body, even a momentary existence independent of matter,—I believe, that when this matter is in a state of repose, mind is perfectly passive to *our cognizance*.

IDA. It is with diffidence, Evelyn, that I enter this arena with a physician, learned in the body; but is there no danger in this doctrine? does it not imply the office of a *gland*,—that brain is the *origin* of soul, and that its function was the *secretion of thought*.

EV. Such is the timid error of the mere metaphysician, Ida. There is no such danger; for, remember, if there *be* secretion, it is the *soul* which *directs*. Many a thought is referred to things which we cannot bring into contact with our consciousness,—*except by the brain.*

Dr. Gall writes of a gentleman, whose forehead was far more elevated on the right side than the left; and he deeply regretted that with this left side he could never think. And Spurzheim, of an Irish gentleman, who has the left side of the forehead the least developed by four lines,—he also could not think with that side, as indeed I have before hinted.

I may tell you the brain is *double*, and *one* healthy *hemisphere* is sufficient, as the organ of mind, if pain or encroachment of the opposite, when diseased, does not destroy life, and this especially when it is a *chronic* change, or exists from birth; so that I have often seen one hemisphere of the brain a pulpy bag of water, and yet vitality and many signs of intellect may still exist; nay, even if the *whole* brain be reduced to one *medullary* bag, *animal* life shall for some time be preserved.

To oppose this blending of mind and matter, Lord Brougham (in his Natural Theology) likens the marble statue hewn into beauty, to the perfect arrangement of organization in a being. While I admire the idea, I may observe that he forgets this truth,—that the maker of the one was a mere *statuary*, without even the fabulous power of Prometheus, or Pygmalion, or Frankenstein; the other, the *Creator* of all things, who *breathed a breath of life into the shape he had made fitted to receive it.* My lord thus halts at the threshold of discovery: mind is not the *product* of organization, but it works *by* and *through* it; and therefore, for its *earthly* uses, cannot be independent of the qualities of matter. We may as well agree with Plato, in endowing the soul with 'a plastic power, to fashion a body for itself, to enter a shape and make it a body living.' I remember Plutarch (in his Quæst. Platon.) makes him say, that the soul is older than the body, and the source of its existence, and that the intellect is in this soul. But where is the sacred evidence of this? for, even in our *antenatal* state, we live, and yet there is probably no consciousness; there is vitality, at least, without the *consciousness* of an intellect.

Astr. As the creation of light was *before* that of the sun, its reservoir, so the creation of the soul might be before the brain, in which the Creator *subsequently* placed it.

Ev. For this there *is* sacred evidence, Astrophel. There *was* light, ere the sun was created as its reservoir; but the soul was breathed into the body, which was already then created.

Astr. This is a specimen of your special pleading, Evelyn, allied to that perilous error of Priestley, that supposed function and structure to be identical, because they are influenced by the same disease, and seem to live and die, flourish and decay, *together*. Democritus also has written his belief that, 'as the smell of a rose exists in the bloom, and fades as that dies, so the soul of an animal is born with its birth, and dies with its death.' You have conceded to me (and we must all be conscious of) the great difficulty of conceiving the *nature* of *spirit;* but, if we are required to prove its existence, we may answer, by analogy, that we cannot always palpably prove the existence of *matter*, although we *know it* to exist. The *electric fluid* may remain for an indefinite period invisible, nay, may never meet the sight,—it may even traverse a space without any evidence but that of its wonderful influence, and at length be collected in a jar.

As light, existing in remote stars, has not yet reached our earth, so the electricity is now residing in myriads of bodies, which will never be elicited; and thus (if I may extend the simile) the principle of life, whatever it be, may have an independent existence during life, may leave the body and yet not perish. Is not this a fine illustration of the living of the soul without the body; for here even a grosser matter, yet invisible, is evinced by its passage from one thing to another, although it is inert when involved in the substance?

Ida. May I not fear that the errors of philosophy, grounded on the difficulty of conceiving the nature of a self-existent spirit, will not stop until they lapse into the belief of anihilation?

For there are many suspicious sentiments even in the pages of well-meaning writers; such are the dangerous sentiments which Boswell has ascribed to Miss Seward:—'There is one mode of the fear of death which is certainly absurd, and that is the dread of annihilation, which is only a pleasing sleep without a dream.'

There may be nothing terrible in the condition of annihilation, yet the moral effect is deplorable; indeed, to doubt the eternal existence is to argue that man's life is but a plaything of the Deity. The notion of annihilation is so abhorrent, that he who believes it dooms himself indeed to a miserable existence; for the crowning solace of a Christian life is holy hope, and belief in the priceless gift of immortality." 186.

High as was our opinion of Mr. Dendy's talents and acquirements, we had no idea of the *extent* of his erudition and researches, till the present work came under our observation. It will assuredly stamp him as one of the most literary characters in the profession at the present time. Although the author, like every man of genius, may have sometimes permitted his imagination to take wide flights, yet the opinions and explanations that flow from Evelyn in this work, are, for the most part, orthodox and scientific.

